# Impact of a dedicated atrial fibrillation clinic on diagnosis-to-ablation time

**DOI:** 10.1016/j.hroo.2022.08.007

**Published:** 2022-09-03

**Authors:** Andrea Robinson, Nagesh Chopra, Auroa G. Badin, Sreedhar R. Billakanty, Keaira Cooper, Eugene Y. Fu, Jennifer James, Victoria Murnane, Jill Swinning, Mitchell Stelzer, Jaret D. Tyler, Anish K. Amin

**Affiliations:** ∗ Section of Cardiac Electrophysiology, Department of Cardiology, OhioHealth Heart and Vascular Physicians, Riverside Methodist Hospital, Columbus, Ohio; † Department of Internal Medicine, OhioHealth Doctors Hospital, Columbus, Ohio

**Keywords:** Atrial fibrillation, Catheter ablation, Diagnosis-to-ablation time, Atrial fibrillation clinic, Risk factor modification

## Abstract

**Background:**

Outcomes following catheter ablation (CA) for atrial fibrillation (AF) improve as the diagnosis-to-ablation time (DAT) shortens. Use of a protocol-based integrated care model through a dedicated atrial fibrillation clinic (AFC) may serve to standardize treatment pathways and decrease DAT.

**Objective:**

To evaluate the DAT and clinical characteristics of patients with AF referred from an AFC vs a conventional electrophysiology clinic (EC).

**Methods:**

Retrospective analysis was completed in consecutive patients undergoing index AF ablation at Riverside Methodist Hospital in 2019 with minimum 1 year follow-up. Patients were categorized based off their CA referral source (AFC vs EC) and where the initial visit following index diagnosis of AF occurred (AFC vs EC).

**Results:**

A total of 182 patients (mean age 65 years, 64% male) were reviewed. Patients referred from an AFC (21%) had a median DAT of 342 days (interquartile range [IQR], 125–855 days) compared to patients referred from EC (79%) with a median DAT of 813 days (IQR, 241–1444 days; *P* = .01). Patients with their index visit following AF diagnosis occurring in the AFC (9%) had significantly shorter median DAT (127 days [IQR, 95–188 days]) compared to EC (91%) (789 days [IQR, 253–1503 days]; *P* = .002). Patients with DAT <1 year had lower AF recurrence than patients with DAT >1 year *(P* = .04, hazard ratio = 0.58, 95% confidence interval 0.3418–1.000).

**Conclusion:**

DAT is a modifiable factor that may affect CA outcomes. Significant reductions in DAT were observed in patients evaluated through a dedicated AF clinic.

**Key Findings**

▪ Diagnosis-to-ablation time (DAT) was reduced by 57.9% in patients with an established history of atrial fibrillation (AF) who were evaluated in a dedicated AF clinic vs an electrophysiology clinic. Further decreases in DAT were appreciated when patients were referred to an AF clinic following initial diagnosis of AF.
▪ DAT less than 1 year vs greater than 1 year resulted in significantly lower AF recurrence.
▪ Our work supports the results of prior studies that suggest DAT is a modifiable factor that may affect catheter ablation outcomes. Significant reductions in DAT were observed in patients cared for in a dedicated AF clinic, suggesting use of a streamlined referral pathway to offer early access to AF specialists can decrease DAT.

## Introduction

Atrial fibrillation (AF) remains an important public health concern, with growing prevalence, increased morbidity and mortality, and an escalating cost to the healthcare system.^1^ Disease management is complicated by the chronic progressive nature of AF and the complex relationship between disease substrate and the coincident comorbid conditions. Unstructured approaches to treating AF lack a comprehensive evaluation and access to management strategies and may further propagate the characteristic structural and arrhythmogenic changes that can limit treatment success.^2^^,^^3^ Atrial fibrillation clinics (AFC) for managing AF have been demonstrated to improve mortality and reduce cost compared to standard care and broaden access to subspecialty care.^4^

Owing to often siloed and segmented care of AF patients across different providers and locations, historical workflows are not designed to provide timely evaluation and escalating treatment strategies for patients. The inability to identify and educate patients early after the diagnosis of AF may be the largest barrier to improving patient outcomes. Antiarrhythmic drug (AAD) therapy and catheter ablation (CA) are safe and effective therapies for treatment of AF.^5^, ^6^, ^7^, ^8^ AFC can standardize the approach to treating AF patients, providing early discussions regarding both rate and rhythm control AF strategies, with an approach geared toward early rhythm control, which has shown to improve clinical outcomes.^9^^,^^10^

Specifically, access to AFC may refine the population of patients referred for CA. In select populations CA has been demonstrated to be superior to AAD therapy as a first line to reduce arrhythmia burden and symptoms. Results of CA may be suboptimal in some patients despite continued improvements in ablation technology and the growing experience of operators.^3^^,^^11^^,^^12^ Poor outcomes following CA may paradoxically lead to further increase in healthcare utilization and reduce patient satisfaction.^13^ CA outcomes are impacted by a number of variables, one of which is the time between initial diagnosis of AF and ablation. Shorter diagnosis-to-ablation time (DAT) consistently improves CA outcomes.^9^^,^^14^, ^15^, ^16^, ^17^, ^18^, ^19^ In our single-center study, we hypothesized that use of a protocol-based integrated-care AFC may optimize treatment and decrease DAT.

## Methods

We completed a retrospective, single-center, observational study of patients undergoing index ablation for paroxysmal (PAF) or persistent (PeAF) atrial fibrillation. Patients were identified using the institutional electrophysiology lab database from January 1, 2019, to December 31, 2019. All patients provided written informed consent prior to the procedure. The protocol was approved by the OhioHealth Research and Innovation institutional review board and the research reported in this paper adhered to the Helsinki Declaration guidelines.

### Study population

Patients were included in the study if they were older than 18 years of age, were undergoing an index ablation for PAF or PeAF as defined by the published guidelines, and were referred from either our AFC or general electrophysiology clinic (EC).^1^^,^^20^ Patients were excluded if they had a prior CA, had an unknown date of AF diagnosis, were enrolled in another clinical trial, had less than 1 year of follow-up, or were referred to CA from outside of our institution (n = 158). A total of 182 patients met inclusion criteria. Patients were categorized based upon where the referral for CA originated, as documented in the electronic medical record (EMR): (1) AFC vs (2) EC. They were also sub-categorized based upon the initial clinic visit following index AF diagnosis having occurred: (1) AFC vs (2) EC. Arrhythmia recurrence was evaluated per the practice standard-of-care monitors at 3 months and 12 months following ablation with duration ranges of 7–30 days, variable per physician. Data from implantable cardiac monitors and electrocardiograms in the EMR were also reviewed, when available, and recurrence was defined as 30 seconds of sustained AF, atrial flutter, or atrial tachycardia. AADs were discontinued following CA based on physician preference.

### AFC model of care

**Figure 1:**
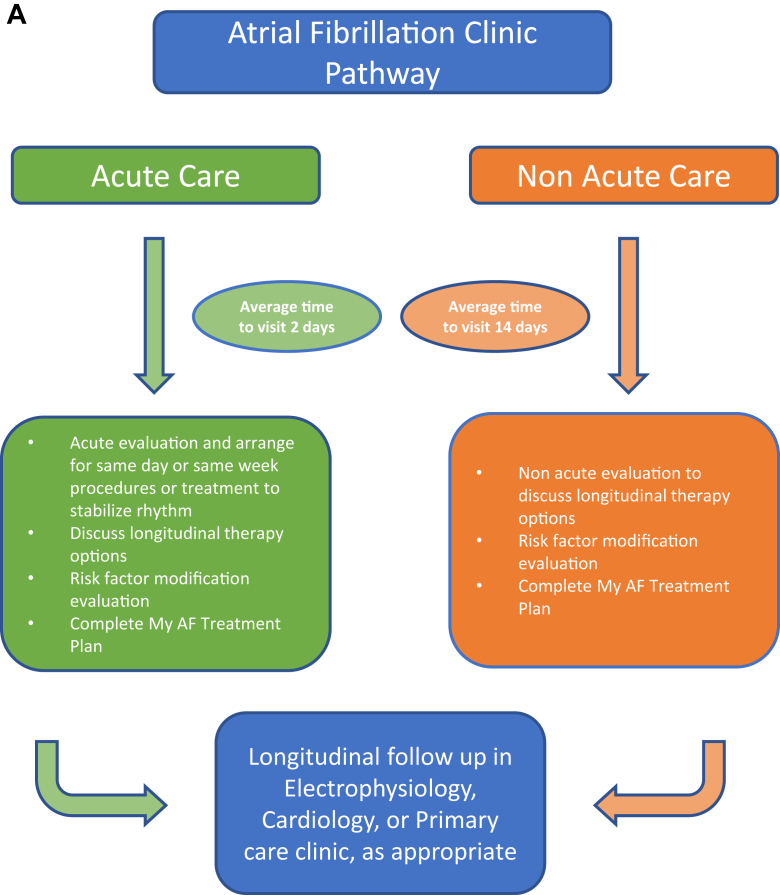

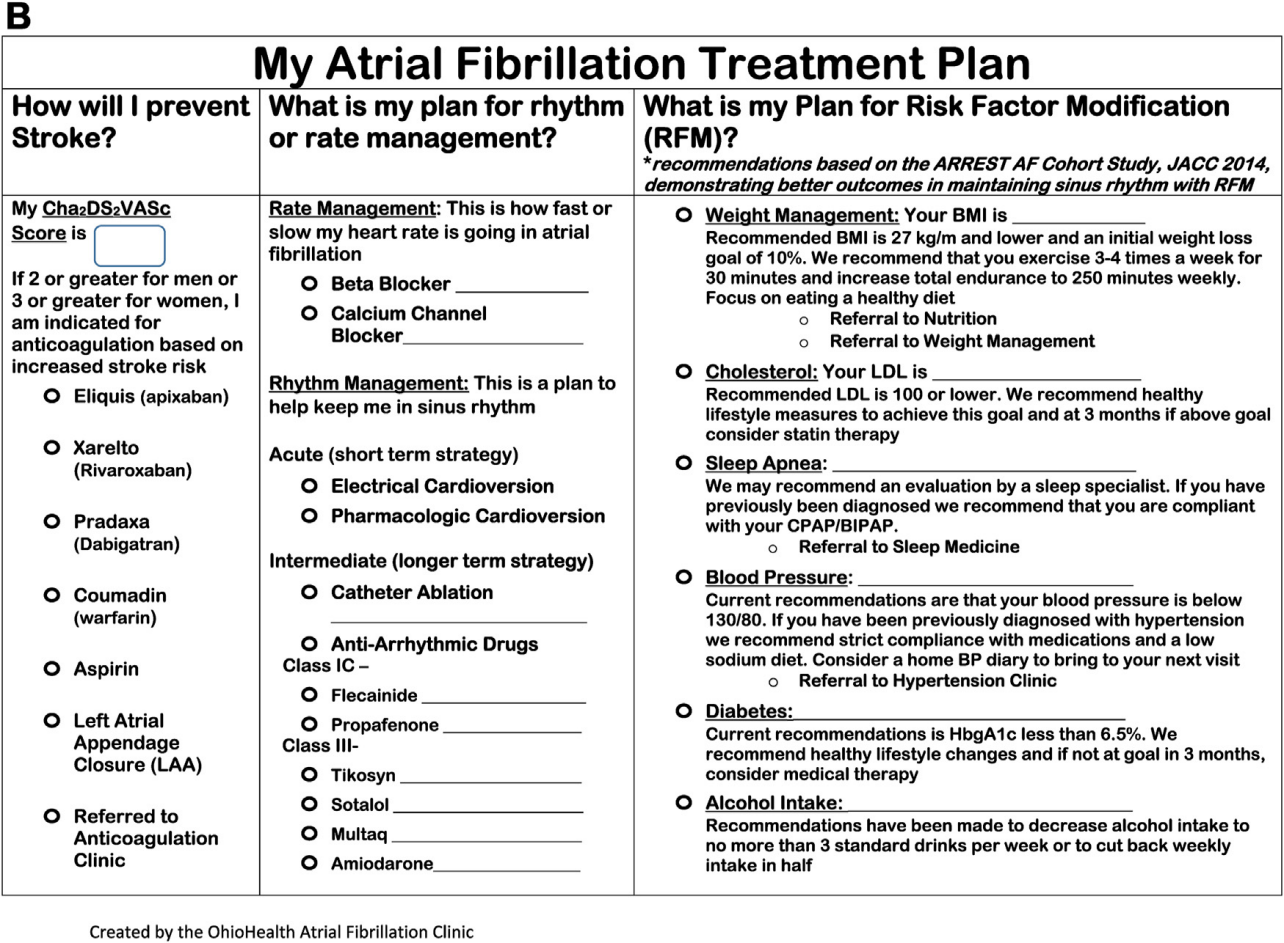
Overview of the atrial fibrillation (AF) clinic. **A:** Flowchart of the acute and nonacute referral pathways into the AF clinic. **B:** Example of the care plan filled out with the patient at each visit.

The AFC is an onsite hospital-based clinic geographically separated from the EC. The goal of the AFC is to facilitate acute outpatient visits and reduce the need for emergency department– and hospital-based evaluations, advance patient engagement and education, and structure the many tenets of AF care. Patients are referred from a variety of paths, including electrophysiology, cardiology, primary care clinics, and the emergency department, for either a new or existing diagnosis of AF, with the majority of visits intended for management of acute episodes of AF (Figure 1A). Patients are seen by an electrophysiology advanced practice provider and a registered nurse, with collaboration of care with an electrophysiologist. To accommodate the comprehensive nature of the visit, each encounter is 50 minutes and includes detailed EMR review to determine prior AF history including date of diagnosis; prior experience with AAD therapy, including adverse effects or intolerances; prior electrical cardioversion and CA history; and an overview of relevant AF risk factors.

#### Acute care pathways

Symptomatic patients referred for an acute evaluation of AF are seen within 2 business days. Examples of patients seen in this pathway include those symptomatic from tachycardia, dyspnea, or heart failure. Therapy options are explored and are inclusive of defining rate vs rhythm control strategies and defining rhythm control strategies of pharmacological cardioversion, electrical cardioversion, AAD therapy, and CA. If clinically indicated, same-day left atrial appendage imaging, pharmacologic and electrical cardioversion, device implant, and/or CA are offered for acute treatment.

#### Nonacute pathways

Asymptomatic patients are seen as routine evaluations and are evaluated on average within 14 business days. Examples of patients seen in this pathway include patients with AF incidentally diagnosed during device interrogation, annual physical, or hospital follow-up from an AF admission. This pathway is indicated to allow a comprehensive conversation and education about AF therapies and accelerate treatment options as clinically indicated.

Education and engagement are central to longitudinal management of AF. To facilitate patient education, patients are asked to participate in their visits by calculating their CHA_2_DS_2_-VASc score and identifying their stroke risk reduction strategy (oral anticoagulant vs left atrial appendage closure). The clinician reviews AF disease physiology using a variety of modalities, including written and verbal descriptions as well as digital applications. Shared decision-making regarding AF treatment approaches as rate vs rhythm control and identifying the medications or interventions facilitating that strategy is completed. Individual risk factors for AF are then reviewed, with goals set for the patient adapted from the ARREST study^21^ and with appropriate referrals placed for specialty care as appropriate. A unique AF treatment plan is ultimately completed at the end of each visit and reviewed with each patient, including strategies for self-care for recurrent episodes (Figure 1B). Patients are asked to follow up longitudinally in the EC but are able to be referred back to AFC as desired for recurrent acute episodes of AF.

### Clinical outcomes and follow-up

As a primary outcome we compared DAT between the AFC and EC groups. DAT was defined as the time elapsed from the first reported date of AF diagnosis to the date of the CA. If the exact day of AF diagnosis was unknown, the first day of the month of the diagnosis was used. The secondary outcome was recurrence of atrial arrhythmia up to 1 year following the date of CA.

Demographic information including comorbid conditions, the type of AF, CHA_2_DS_2_-VASc score, and clinical characteristics were determined by reviewing the EMR in the visit prior to the CA. Evaluation of risk factor modification (RFM) counseling and referral for specialty evaluation were determined by documentation and referral orders in the EMR. Study data were collected and managed using REDCap electronic data capture tools hosted at OhioHealth.

### Statistical analysis

Mean and standard deviation (for normally distributed variables) or median and 25th and 75th percentile (for variables not normally distributed) were computed for continuous variables. For categorical variables, proportion and frequency count were calculated. Group comparisons of categorical variables were made using Fisher exact or χ^2^ test and of continuous variables using Student *t* test (for normally distributed variables) and Mann-Whitney *U* test (for variables not distributed normally). Cox regression analysis was used to explain the relationship between a dependent binary outcome and a continuous or categorical independent variable where indicated. Kaplan-Meier analysis with Gehan-Breslow-Wilcoxon test was used to analyze AF recurrence after ablation in the early referred (<1 year) vs late referred (>1 year) groups. A statistical test was considered significant if the *P* value was <.05. GraphPad statistical software Prism 9 Version 9.1.2 was used for statistical analysis of the data.

## Results

Of the 340 patients undergoing AF CA at our institution from January 1 to December 31, 2019, 182 met all inclusion and exclusion criteria. The patient demographics are summarized in Table 1. The mean age was 65 ± 11 years of age and 36% were female. The mean left ventricular ejection fraction was 57% ± 10%; 133 (73.1%) patients were paroxysmal, 48 (26.4%) patients were persistent, and 1 patient (0.5%) had long-standing PeAF. The mean left atrial size was 4.2 ± 0.7 cm and the mean body mass index was 32.8 ± 7.23. Mean CHA_2_DS_2_-VASc score was 2.7 ± 1.7. Observed comorbidities included hypertension (75.8%), coronary artery disease (22%), and diabetes mellitus (22.5%). Sleep apnea was a reported diagnosis in 35.7% of patients.

**Table 1:** Baseline characteristics of the overall study population and categorized by referral source

Variable	All patients	Electrophysiology clinic	Atrial fibrillation clinic	*P* value
N	182	144	38	
Age, y	65.2 ± 10.8	65.3 ± 10.9	65.1 ± 10.3	.93
Female sex	36.3	36.8	34.2	.77
BMI, kg/m^2^	32.8 ± 7.23	32.7 ± 7.4	33.1 ± 6.7	.72
Coronary disease	22	21.5	23.7	.78
Hypertension	75.8	77.8	68.4	.23
Diabetes mellitus	22.5	22.2	23.7	.85
Sleep apnea	35.7	35.4	36.9	.87
CHA_2_DS_2_-VASc score	2.73 ± 1.77	2.76 ± 1.84	2.60 ± 1.48	.62
LVEF, %	57.1 ± 9.7	56.5 ± 10.2	59.2 ± 7.0	.07
Left atrial size, cm	4.2 ± 0.7	4.2 ± 0.7	4.3 ± 0.8	.5
Paroxysmal AF	73.1	76.4	60.5	.05
Ablation				
Cryo	39	40.3	34.2	.5
RFA	61	59.7	65.8	.5
PV isolation	100	100	100	
CTI line	56	56.9	52.6	.63
Additional ablation	13.2	12.5	15.8	.59

Numbers are % or mean ± SD. AF = atrial fibrillation; BMI = body mass index; Cryo = cryoballoon; CTI = cavotricuspid isthmus; LVEF = left ventricular ejection fraction; PV = pulmonary vein; RFA = radiofrequency ablation.

### Diagnosis-to-ablation time

**Figure 2:**
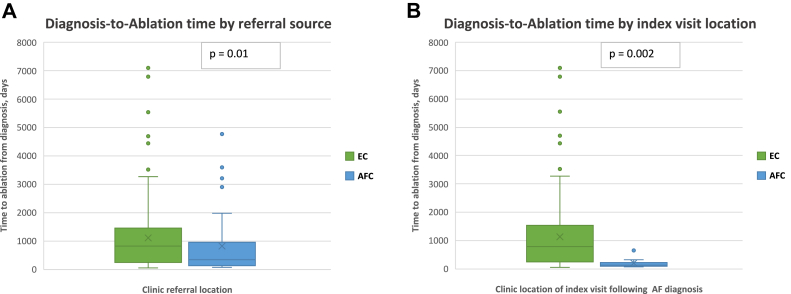
Diagnosis-to-ablation time (DAT) in this patient cohort. **A:** DAT evaluated based on the location of the referral for ablation. **B:** DAT evaluated based on the location of the first clinic visit following initial diagnosis of atrial fibrillation. AFC = atrial fibrillation clinic; EC = electrophysiology clinic.

**Figure 3:**
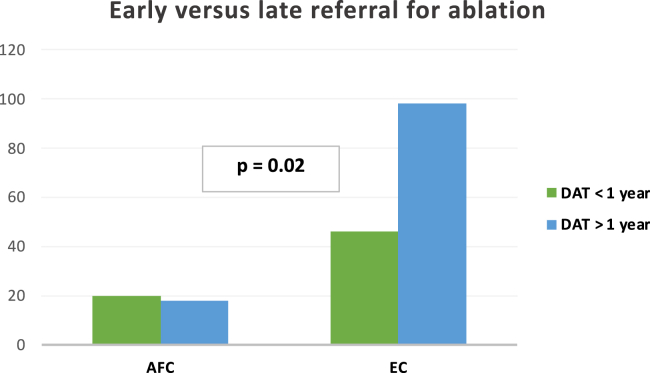
Early vs late diagnosis-to-ablation time (DAT) by referral source (N =182). AFC = atrial fibrillation clinic; EC = electrophysiology clinic.

Thirty-eight patients (21%) were referred from a dedicated AFC and had a median DAT of 342 days (interquartile range [IQR], 125–855 days) compared to 144 patients referred from EC (79%) with a median DAT of 813 days (IQR, 241–1444 days; *P* = .01). Of the patients referred from the AFC, 17 of 38 (45%) were evaluated through the acute pathway. Following initial AF diagnosis, 16 patients (9%) had their index visit in AFC, compared to 166 patients (91%) having their visit at EC. Median DAT was significantly shorter following AFC visit (127 days [IQR, 95–188 days], compared to 789 days in the EC [IQR, 253–1503 days]; *P* = .002; Figure 2). One hundred and thirty-three patients had PAF and demonstrated a median DAT of 654 days (IQR, 179–1294 days). Of the 23 (17.2%) patients with PAF referred from AFC, median DAT was 235 days (IQR, 122–623 days), vs 813 days (IQR, 223–1438 days) (*P* = .002) in the 110 patients with PAF (82.7%) referred from EC. Forty-nine patients with PeAF demonstrated a mean DAT of 694 days (IQR, 250–1478 days). DAT in this AF subtype based upon referral source, AFC (15 patients, 31%) vs EC (34 patients, 69%), was 520 days (IQR, 147–1831 days) vs 810 days (IQR, 275–1431 days) (*P* = .68). Sixty-six patients had a DAT of less than 1 year; 30% were referred from the AFC vs 70% from the EC. A significantly higher number of patients had DAT <1 year when evaluated at the AFC in comparison to the EC (52% vs 32%; *P* = .02) (Figure 3), and patients with DAT <1 year were younger (62.2 vs 66.8 years, *P* = .01) and less likely to have hypertension (Table 2).

**Table 2:** Clinical variables dichotomized based on diagnosis-to-ablation time

Variable	DAT (<1 year)	DAT (>1 year)	*P* value
N	66	116	
Age (years)	62.2 ± 11.7	66.8 ± 9.8	.01
Female sex (%)	20 (30)	46 (40)	.27
BMI kg/m^2^	33.1 ± 7.2	32.5 ± 7.2	.48
Coronary disease	12 (18)	28 (24)	.45
Hypertension	43 (65)	95 (82)	.01
Diabetes mellitus (%)	17 (26)	24 (21)	.46
Sleep apnea	23 (35)	42 (36)	.87
LVEF, %	55.9 ± 10.4	57.7 ± 9.2	.34
Left atrial size, cm	4.2 ± 0.8	4.2 ± 0.7	.99
Paroxysmal AF	51 (77)	82 (71)	.48
Ablation			
Cryo	22 (33)	49 (42)	.27
RFA	44 (67)	67 (58)	.27
PV isolation	66 (100)	116 (100)	ns
CTI line	36 (55)	66 (57)	.75
Additional ablation	8 (12)	16 (14)	.82
AF clinic as referral source for ablation	20 (30)	18 (16)	.02
DAT (median [25–75] percentile)	146 [107–222]	1137 [692–1979]	<.0001

Numbers are % or mean ± SD.

AF = atrial fibrillation; BMI = body mass index; Cryo = cryoballoon; CTI = cavotricuspid isthmus; DAT = diagnosis-to-ablation time; LVEF = left ventricular ejection fraction; ns = nonsignificant; PV = pulmonary vein; RFA = radiofrequency ablation.

### Ablation outcomes

**Figure 4:**
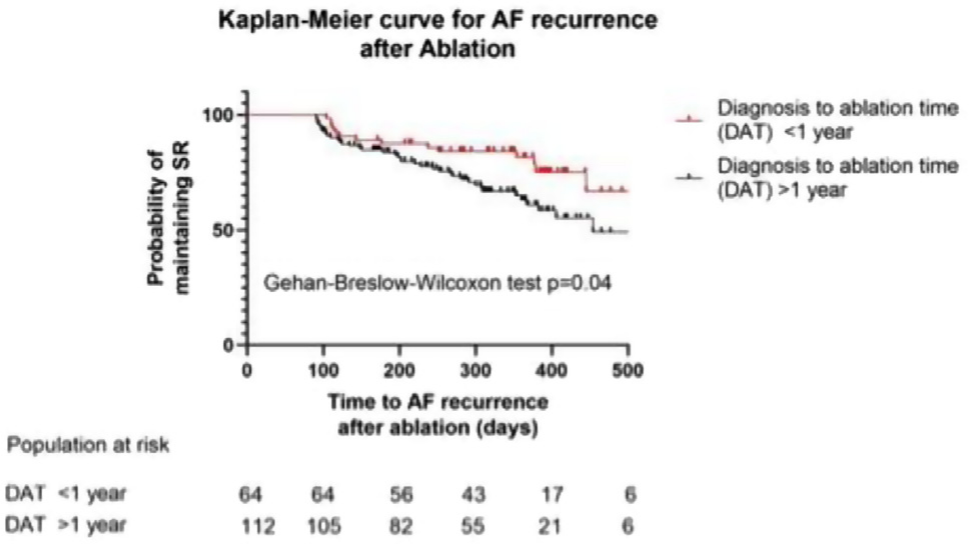
Kaplan-Meier curve for atrial fibrillation (AF) recurrence after ablation. Patients referred for AF ablation with a diagnosis-to-ablation time (DAT) <1 year are significantly more likely to maintain sinus rhythm than those with a DAT >1 year.

AF recurrence occurred in 60 out of 182 (33%) patients during 1 year of follow-up. Of the 77% (140/182) of patients on AAD at the time of CA, 34% (62/182) remained on drug therapy at 12 months. A Cox regression analysis was performed to evaluate the individual contribution of several independent clinical variables that may affect AF recurrence after AF ablation (Table 3). Recurrence by referral source was 46 of 144 (32%) in the EC group and 14 of 38 (37%) in the AFC group (*P* = .56). Left atrial size and DAT were independently associated with AF recurrence. For every 1 cm increase in LA size there was a 47% increase in risk of AF recurrence following AF ablation over the study period *(P =* .04). For every 12-month delay in AF ablation after diagnosis there was a 9.4% increased risk of AF recurrence following AF ablation over the study period *(P =* .007). Diagnosis to ablation time less than 1 year vs greater than 1 year resulted in significantly lower AF recurrence (Figure 4) *(P =* .04, hazard ratio = 0.58, 95% confidence interval 0.3418–1.000).

**Table 3:** Adjusted hazard ratios of clinical variables influencing atrial fibrillation recurrence after atrial fibrillation ablation using Cox regression analysis

Clinical variable	Hazard ratio	95% confidence interval	*P* value
Left atrial size (cm)	1.48	1.008–2.128	.037
DAT (years)	1.094	1.017–1.165	.008
Type of AF (PAF vs PeAF)	1.439	0.731–2.722	.274
Energy source used for AF ablation (cryo vs RF)	0.993	0.508–1.980	.984
Presence of cardiomyopathy	0.895	0.376–1.895	.785
Source of referral (dedicated AF clinic vs conventional EP clinic)	1.524	0.698–3.130	.266

AF = atrial fibrillation; Cryo = cryoballoon; DAT = diagnosis-to-ablation time; EP = electrophysiology; PAF = paroxysmal atrial fibrillation; PeAF = persistent atrial fibrillation; RF = radiofrequency.

There were no significant differences between the AFC and the EC patients with respect to AAD therapy use, age, CHA_2_DS_2_-VASc score, or body mass index.

### Risk factor modification

RFM evaluation was considered to have been completed when there was a documented encounter in the progress note that evaluated and reviewed recommendations for hypertension, diabetes, dyslipidemia, sleep apnea, obesity, and use of alcohol, as applicable, to each patient. Of the 38 patients in the AFC cohort, 36 (95%) underwent EMR-documented RFM counseling, compared to 57 (40%) in the EC cohort (*P <* .00001). The proportion of patients referred for sleep apnea evaluation and therapy in patients without a previous diagnosis in the AFC cohort was 50% (19 patients) compared to 20% (36 patients) in the EC (*P* = .003). The outcomes of the effect of the RFM counseling was not assessed in this retrospective analysis.

## Discussion

This single-center retrospective evaluation of 182 patients undergoing AF ablation is the first demonstration, to our knowledge, of the impact of an AFC on reducing DAT. Diagnosis to ablation time was reduced by 57.9%, 813 days to 342 days, when patients with an established history of AF were evaluated in an AFC vs conventional EC. Patients evaluated following an index diagnosis of AF in an AFC had further reduction in DAT: 789 days to 127 days, 83.9%. A higher proportion of patients referred for ablation from AFC vs EC had a DAT of less than 1 year. Diagnosis to ablation time of less than 1 year significantly reduced AF recurrence.

AF is the most prevalent arrhythmia, with tremendous downstream healthcare resource utilization. Late-stage complications of stroke and heart failure as well as hospitalization drive the majority of morbidity and mortality and subsequent healthcare costs. Early management of AF, focused on a rhythm control strategy, has been demonstrated to reduce hospitalization and stroke.^10^^,^^22^^,^^23^

AF ablation as a first-line intervention has been demonstrated to yield superior results in arrhythmia recurrence compared to drug therapy.^24^, ^25^, ^26^, ^27^ The rationale for early intervention arises from the complex interaction between the direct injury and remodeling on atrial structures induced by AF and the comorbid conditions that associate with AF. The presence of AF directly promotes hemodynamic strain and inflammation, coupled with the impact of traditional risk factors such as hypertension, sleep apnea, obesity, and diabetes; an acceleration of fibrosis ensues, underpinning the progressive nature of the disease. Early interventional strategies interrupt this process, resulting in reduced arrhythmia burden following ablation, compared to drug therapy, and decreased healthcare resource utilization. Debate surrounding the optimal ablation lesion set for the various stages of AF undervalues the consistent predictor of ablation outcomes, time from diagnosis to ablation. The maximal benefit of ablation seems to occur early in the disease state when diffuse remodeling of the atrial structures is minimal. Identifying barriers to access ablation and modifiable factors that influence ablation success is paramount, as increasing evidence supports early ablation.

Diagnosis to ablation time is an independent factor in determining ablation success.^14^, ^15^, ^16^, ^17^, ^18^, ^19^ DAT of <1 year has particular correlation with outcomes. De Greef and colleagues^17^ demonstrated superior ablation outcomes in 1000 patients with 5-year follow-up with DAT <1 year in patients with primarily PAF. Patients with PeAF were studied by Hussein and associates,^16^ with similar findings at 2-year follow-up, with the lowest AF recurrence occurring when DAT was <1 year. These data are independent of the ablation strategy that was undertaken, reinforcing the importance of early referrals when considering interventional strategies. A more recent study by Chew and colleagues^19^ analyzed a large nationwide cohort of 11,143 patients and found that for every 1 year increase in DAT, risk for AF recurrence after CA is increased by 20%. Our results are consistent with these findings. Our experience differs by employing a streamlined referral pathway to offer early access to a specialist provider, which decreases time to interventional therapy. In patients with a preexisting diagnosis of AF, AFC care reduces DAT in both paroxysmal and persistent cohorts. This effect was magnified when initial evaluation following first diagnosis of AF was completed in an AFC compared to an EC. Overall, a clinical endpoint of DAT <1 year was realized in patients referred from an AFC vs an EC.

The safety and efficiency of ablation solutions has evolved exponentially, yet the requisite resources to expand access and awareness for rhythm-based strategies has not followed a similar trajectory. Comprehensive AF management requires patient engagement and serial mechanisms to minimize healthcare resources and hospitalization for breakthrough arrhythmias as well as promoting RFM.^28^ AF pathways facilitate and improve the care of AF patients. These pathways are superior to standard practice in evaluating and prescribing anticoagulation as well as lowering cost of care for this patient population. Hendriks and colleagues^4^ demonstrated a 48% reduction in hospitalization for patients cared for through registered nurse–led AF pathways relative to standard care and offered a significant mortality advantage at 3 years.

Advanced practice provider–led AF pathways allow for streamlined escalation of therapy. Access to expert care in a timely manner provides the opportunity to educate patients regarding the full breadth of treatment options, including and not limited to rate control options vs rhythm control with either AAD or intervention. Our AFC pathway provides 50-minute visits, compared to the 20-minute visits standard in the EC. This allows clinicians to conduct in-depth discussions and to use digital applications to educate patients, which may not be feasible in a shorter clinic appointment. Facilitating patient education and removing referral bias expands opportunities to maximize evidence-based care and specifically extend interventional solutions as an option to our patients in the setting of shared decision-making. Our experience demonstrates the role of an advanced practice provider–led AFC on accelerating DAT.

### Limitations

Several limitations were observed. Defining the exact date of diagnosis in some patients required looking at historical EMR databases and was sometimes based on a subjective description in progress notes. The temporal pattern and duration of AF on a patient level was not readily available in this retrospective series. Recurrence rate of AF was reviewed in a retrospective observational fashion based on documentation on electrocardiograms, external cardiac monitors, and implantable cardiac devices and may be underestimated. A majority of the follow-up period following CA occurred during the COVID-19 pandemic, which may have limited in-person visits, may have increased use of telehealth, or could have confounded results of atrial arrhythmia recurrence with an unknown correlation from factors related to the worldwide pandemic. Long-term impact on AF recurrence may be impacted by the relatively small patient sample size. This is a single-center study demonstrating decreased DAT using a referral pathway, which may not be realized in all centers owing to complexities of specific logistics with scheduling ablation following referral. The logistics of completing an AF ablation in many labs may be a larger hurdle in delivering care.

## Conclusion

Patients receiving care through a dedicated AFC demonstrate shorter diagnosis to ablation times, particularly if the index visit following an initial AF diagnosis occurs in the AFC. Coordinated care in an AFC results in superior referral for risk factor management. AFCs expand patient access and facilitate the longitudinal management of AF. Further studies will be needed to generate more evidence around optimal AF workflows. Future trials can, we hope, improve on the limitations of traditional workflows relative to disease-specific clinics.
